# Ayahuasca partially preserves striatal integrity in juvenile non-human primates exposed to chronic stress: evidence from stereological evaluation

**DOI:** 10.3389/fnana.2025.1457557

**Published:** 2025-02-19

**Authors:** Wigínio Gabriel Lira-Bandeira, Lílian Andrade Carlos Mendonça Batista, Andréa Silva Medeiros-Bandeira, Paulo Leonardo Araújo Góis Morais, Luiz Roberto Fernandes Pereira, Maria Lara Porpino Meiroz-Grilo, Jeferson Souza Cavalcante, Melquisedec Abiaré Dantas Santana, Ruthnaldo Rodrigues Melo Lima, Nicole Leite Galvão-Coelho, Fernando Vagner Lobo Ladd, Expedito Silva Nascimento

**Affiliations:** ^1^Laboratory of Neuroanatomy, Department of Morphology, Biosciences Center, Federal University of Rio Grande do Norte, Natal, RN, Brazil; ^2^Laboratory of Experimental Neurology, Department of Biomedical Sciences, State University of Rio Grande do Norte, Mossoró, RN, Brazil; ^3^Laboratory of Hormone Measurement, Department of Physiology and Behavior, Biosciences Center, Federal University of Rio Grande do Norte, Natal, RN, Brazil; ^4^Laboratory of Neurochemistry, Department of Physiology and Behavior, Biosciences Center, Federal University of Rio Grande do Norte, Natal, RN, Brazil

**Keywords:** caudate, putamen, ayahuasca, striatum, depression, chronic stress, stereology

## Abstract

**Introduction:**

The striatum (St) integrates cognitive, motor, and limbic functions and plays a critical role in processing emotions, motivation, and rewards. It may undergo several morphophysiological changes in neuropsychiatric diseases. Depression, a complex psychiatric disorder, affects millions of people around the world and leads to an increased risk of suicide, decreased quality of life, and functional impairment. Conventional treatments require prolonged use, leading to drug resistance; thus, new treatments and therapeutic strategies have been widely studied. Ayahuasca results from the joint infusion of the *Banisteriopsis caapi* vine and Psychotria viridis leaves have psychoactive properties, and its use in depression has shown promising results. Our objective was to morphoquantitatively evaluate the effects of ayahuasca on the St in an already validated model of juvenile depression induced in a non-human primate.

**Methods:**

Six marmosets were divided into three groups of two animals each. One group was kept in family life (FG), and two groups were socially isolated (IG). Isolation was carried out by separating the animal from all others in the colony. One of the isolated groups received doses of ayahuasca tea (AG) 3 days before and two times during the isolation period, while the other groups received the same dose of placebo. After 13 weeks of experimentation, euthanasia, and transcardiac perfusion were performed. The brains were sectioned and stained with thionin using the Nissl method. We employed stereological techniques to assess the striatum and investigate potential alterations in neuronal volume in socially isolated animals treated with ayahuasca. Equidistant sections of the caudate and putamen were analyzed for all measurements and selected by systematic and uniform sampling.

**Results and discussion:**

Striatal neurons in the IG group exhibited significantly smaller volumes compared to those in the FG and AG groups. Our findings suggest that ayahuasca may prevent extensive neuronal volume loss, as observed in the IG, by acting as a prophylactic agent and buffering neural structural changes during chronical social isolation.

## Highlights

- Social isolation decreases the volume of striatal neurons.
- Striatal neurons have adaptative stress response in juvenile marmosets administrated with ayahuasca.
- Ayahuasca demonstrated a potential prophylactic effect by enhancing the resilience of striatal neurons against volume loss during chronic social isolation.

## 1 Introduction

Depression is a debilitating mental health condition affecting approximately 280 million individuals globally, including an estimated 5% of adults and 5.7% of elderly people over the age of 60 (WHO, 2023). Its etiology and treatment involve a complex interplay of genetic, neurobiological, psychological, and environmental factors, posing a significant challenge to scientific understanding and effective clinical intervention (Marx et al., 2023). Among the various brain regions implicated in the pathophysiology of depression, the striatum emerges as a crucial component of this complex neurobiological puzzle (Xu et al., 2020).

The striatum (St), located in the basal region of the brain, is traditionally associated with motor control (Graybiel, 2008). However, increasing interest surrounds its role in cognitive and emotional processes (Graybiel, 2008; Provost et al., 2015). Anatomically composed of the caudate nucleus (Cd) and putamen (Pu) (Alexander and Crutcher, 1990), the St’s functional significance extends beyond motor functions, encompassing participation in circuits regulating mood, motivation, and behavior (Basile et al., 2021).

The St serves as the primary input center of the basal ganglia (Shepherd, 2013). In addition to receiving projections from subcortical areas such as the thalamus, amygdala, hippocampus, and substantia nigra pars compacta, the St receives extensive input from cortical regions (Choi et al., 2019; Miura et al., 2020). Notably, projections from the limbic and prefrontal cortices suggest a crucial role for the St in regulating emotions, motivation, and decision-making (Shepherd, 2013). This convergence of fibers positions the St as a strategic target for investigations into the neurobiology of mood disorders, including depression.

The understanding of the neurobiological mechanisms underlying depression has expanded considerably in recent decades, with significant insights emerging regarding chronic stress as a precursor to various clinical conditions and the growing appreciation for the role of corticostriatal circuits (LeMoult et al., 2023). These circuits, which connect cortical areas with the St, play a crucial role in regulating mood, motivation, and behavior (Siemsen et al., 2022). Dysfunctions in these circuits have been implicated in a range of psychiatric disorders (Lee et al., 2022), including depression. As a critical hub within these circuits, the St is emerging as a promising target for further investigation into the neurobiological bases of chronic stress and depression (Shepherd, 2013).

Advances in neuroimaging and neuroanatomical techniques have revealed structural and functional changes in the St of individuals with depression (Han et al., 2023). Studies in humans and animals suggest that striatal dysfunctions play a crucial role in the pathogenesis of depression (Qi et al., 2018; Avnioglu et al., 2021). Abnormalities in the connectivity and synaptic plasticity of these nuclei may contribute to depressive symptoms (Xu et al., 2020; Rodrigues et al., 2022), including anhedonia, apathy, and executive dysfunction.

The relationship between depression and the St becomes even more significant when considering potential treatments. Currently, medicaments considered the gold standard for treating depression include second-generation antidepressants, such as selective serotonin reuptake inhibitors and serotonin-norepinephrine reuptake inhibitors (Kovich et al., 2023). Fluoxetine hydrochloride, a widely prescribed SSRI, is often the first-line treatment for children, adolescents, and young adults (Hussain et al., 2018).

The medications currently used often take around 2 weeks to show significant improvements (Cipriani et al., 2018), potentially leading to chronic use (Harmer et al., 2017) and, in many cases, antidepressant resistance (Ho and Zhang, 2016). In this context, studies on the effects of psychoactive substances, such as ayahuasca, have emerged. Ayahuasca is a brew prepared from the *Banisteriopsis caapi* vine and *Psycotria viridis* leaves. Its primary psychoactive compound, N, N-dimethyltryptamine (DMT), has potent psychedelic properties (Perkins et al., 2022; Rossi et al., 2022). This compound, found in various plants, is used in traditional rituals in some cultures and has recently garnered interest for its potential antidepressant and anti-inflammatory effects (Osório Fde et al., 2015; Palhano-Fontes et al., 2019). DMT acts as a serotonin (5-HT) receptor agonist, modulating neuronal activity (Carbonaro and Gatch, 2016; Luan et al., 2024)_._

Most research on mood disorders focuses on adult or early childhood animal models, with relatively few studies investigating the juvenile period. However, the juvenile phase is a crucial ontogenetic stage, marked by remarkable plasticity within the nervous system. This plasticity makes the juvenile brain particularly sensitive to environmental influences, which can induce lasting changes in cognition and the stress response system. Adverse experiences during this critical period can lead to enduring cognitive, behavioral, and physiological problems in adulthood, increasing the risk of mood disorders such as depression (Hankin, 2006; Ganzel and Morris, 2011; Thapar et al., 2012). In recent years, the incidence of depressive episodes in adolescents has increased (Qin et al., 2015), affecting approximately 14% of young people between 15 and 18 years old. Furthermore, there is a recurrence rate of approximately 40% within 3–5 years following the first episode (Hankin, 2006).

Previous studies conducted by our team (Galvão-Coelho et al., 2017; da Silva et al., 2018) have validated chronic social isolation as an effective protocol for inducing depression and hypocortisolemia in juvenile marmosets. Additionally, ayahuasca has been shown to act as a prophylactic substance, preventing some depressive symptoms (de Meiroz Grilo et al., 2022). However, few studies have investigated the effects of ayahuasca in animal models of depression, and there is a particular gap in knowledge regarding how this substance affects the morphology of the striatum. This gap limits our understanding of the potential risks and benefits of ayahuasca and hinders the identification of possible health repercussions for individuals.

This study aimed to investigate the morphoquantitative effects of ayahuasca on the St in a non-human primate model of depression. We employed stereological techniques to advance the understanding of the pathophysiology of depression and contribute to the development of novel therapeutic approaches for this debilitating condition. This research has significant implications for improving individuals’ mental health and wellbeing worldwide.

## 2 Materials and methods

This research thoroughly analyzed serial coronal brain sections from six marmosets. All research protocols were carried out in accordance with the recommendations of the Federal University of Rio Grande do Norte (UFRN) in the Animal Experimentation Ethics Committee (approved under protocol no. 225.001/2020 and addendum 5 of no. 032/2014).

### 2.1 Experimental depression model

The common marmoset (*Callithrix jacchus*) is a small neotropical primate that is easy to handle and adaptable to captive environments. It is currently classified as a species of “Least Concern” on the Red List of Threatened Species of the International Union for Conservation of Nature (Miranda et al., 2015).

Due to its close phylogenetic relationship with other primates, including humans, the marmoset has become a prominent model organism in studies focused on chronic stress. It is a validated animal model for investigations related to depression, as it exhibits physiological and behavioral changes that resemble those observed in humans with depression when exposed to chronic stress paradigms (Pryce et al., 2002; Galvão-Coelho et al., 2008; de Sousa et al., 2021; de Meiroz Grilo et al., 2022).

### 2.2 Experimental design

This study utilized brain samples collected from the study conducted by de Meiroz Grilo et al. (2022). The animals were raised at the UFRN Primatology Center and housed under natural lighting conditions with controlled temperature and humidity, with food and water provided ad libitum. The analysis of the results involved a total of six marmosets distributed into three groups: Family Group (FG), Isolated Group (IG), and Ayahuasca Group (AG). At the beginning of the experiment, all animals were aged between 7 and 9 months, classified as juveniles I and II (de Castro Leão et al., 2009), and reached the end of the experiment, aged between 11 and 13 months. The animals in the FG served as the control group, while the IG animals acted as a negative control group. The AG subjects constituted the experimental group.

The study followed the protocol for inducing depression due to chronic stress established by Galvão-Coelho et al. (2017). It was divided into two distinct stages: the basal phase and the experimental phase. In the basal phase, all animals were housed socially with their families for 4 weeks. After this phase, the FG (*n* = 2) animals remained in the same conditions for another 9 weeks. The experimental phase consisted of total social isolation from other animals for 9 weeks. In this second stage, the IG (*n* = 2) and AG (*n* = 2) animals were separated from their families and kept in individual cages, completely isolated from other animals, without visual contact or proximity between their families’ accommodation enclosures. Prolonged exposure to social isolation is a significant chronic stressor and is known to induce depressive-like behaviors in marmosets (Galvão-Coelho et al., 2017; da Silva et al., 2018).

**Figure 1:**
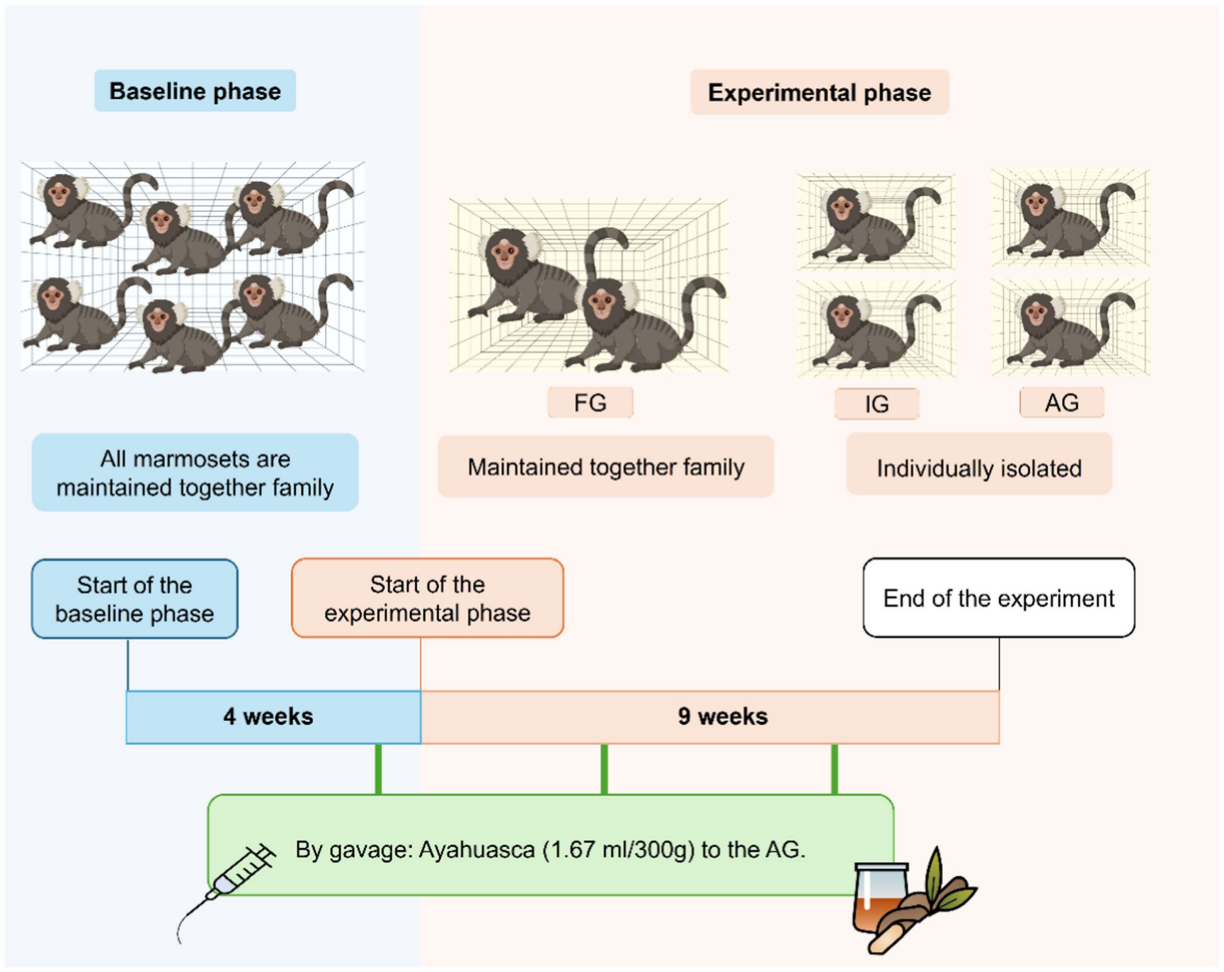
Schematic drawing of the experimental design. On the left side of the image, in blue, Basal Phase events: all animals maintained in family interaction, and 3 days before the end of this phase, the animals received, by gavage, Ayahuasca for AG animals. In orange, the right side of the image shows events from the Experimental Phase: animals from groups IG and AG are subjected to social isolation, and FG continues in family interaction. Below is a green timeline of the treatment administrations with Ayahuasca for AG animals. The first gavage occurred 3 days before the end of the baseline phase, and two more administrations occurred during the experimental phase, 25 and 50 days after the first gavage.

A total of three doses of ayahuasca tea (1.67 mL/300 g) were administered by oral gavage to the AG animals, constituting a preventive and chronic treatment (Galvão et al., 2018; Palhano-Fontes et al., 2019). These administrations were carried out 3 days before isolation, and two more doses were made 25 and 50 days after the first (Figure 1).

The dosage used was based on previous research in which 1 mL of ayahuasca was administered per kilogram of body weight, similar to its use in traditional Brazilian rituals. Allometric scaling, considering the differences in metabolic rates described by Pachaly (2024), was applied to adjust the dosage for the marmosets. The basal metabolic rate of a typical 70 kg human was calculated as 1,694 kcal, while that of a 300 g marmoset was calculated as 28.38 kcal. Thus, the dose of ayahuasca was determined to be 0.06 mL/kcal for humans and adjusted to 1.67 mL for marmosets (de Sousa et al., 2021).

A single batch of ayahuasca, prepared by Templo da Barquinha in Ji-Paraná, Rondônia, was used for this study. The brew was prepared with *P. viridis* leaves (50%) and *B. caapi* bark (50%), boiled in water for 60 h. Water evaporation was compensated for by adding more water, bark, and leaves. The final batch was stored in glass jars in a refrigerator (da Silva et al., 2018). The concentration of the main alkaloids was quantified by mass spectroscopy, showing 0.36 ± 0.01 mg/mL of DMT, 1.86 ± 0.11 mg/mL of harmine, 0.24 ± 0.03 mg/mL of harmaline, and 0.20 ± 0.05 mg/mL of THH (Savoldi et al., 2017; Palhano-Fontes et al., 2019).

Ayahuasca was administered by oral gavage using flexible, lubricated cannulas to minimize the risk of damage to the gastrointestinal tract. The animals were fed small portions of fruit before the procedure, which was carried out 15 min after feeding. All animals were previously adapted to the method and handling to minimize stress (da Silva et al., 2018).

### 2.3 Euthanasia, perfusion, microtomy, and staining technique

At the end of the experiment, the animals were euthanized with sodium thiopental (Abbott, São Paulo, SP, Brazil; 40 mg/kg, i.v.) according to the protocols and doses approved by the UFRN Institutional Animal Care and Use Committee, which consist of three times the sedation dose administered intraperitoneally.

Transcardiac perfusion was performed via the left ventricle and right atrium using a three-phase perfusion pump. The first phase involved a saline solution with an anticoagulant infused for 5 min at a high flow rate to clear the vascular bed. The second phase consisted of a 4% paraformaldehyde solution in 0.1 M phosphate buffer (pH 7.4) infused for 45 min at a slow flow rate. Finally, a solution of 4% paraformaldehyde and 15% sucrose in 0.1 M phosphate buffer (pH 7.4) was infused for 30 min at a slow flow rate.

After perfusion, the animals were decapitated, and their brains were extracted via craniotomy. The brains were post-fixed in 4% paraformaldehyde overnight in 0.1 M phosphate buffer (pH 7.4). They were then cryoprotected in a solution of 4% paraformaldehyde and 15% sucrose in 0.1 M phosphate buffer (pH 7.4) for 3 days, followed by immersion in a 30% sucrose solution in 0.1 M phosphate buffer (pH 7.4) until microtomy.

Six parallel series of 40 μm-thick coronal sections were obtained using a cryostat at −20°C. The brain tissue was embedded in Tissue-Tek blocks and subjected to rapid freezing before sectioning. The first series of sections was mounted on positively charged slides for Nissl staining with thionine.

### 2.4 Stereological data collection

**Figure 2:**
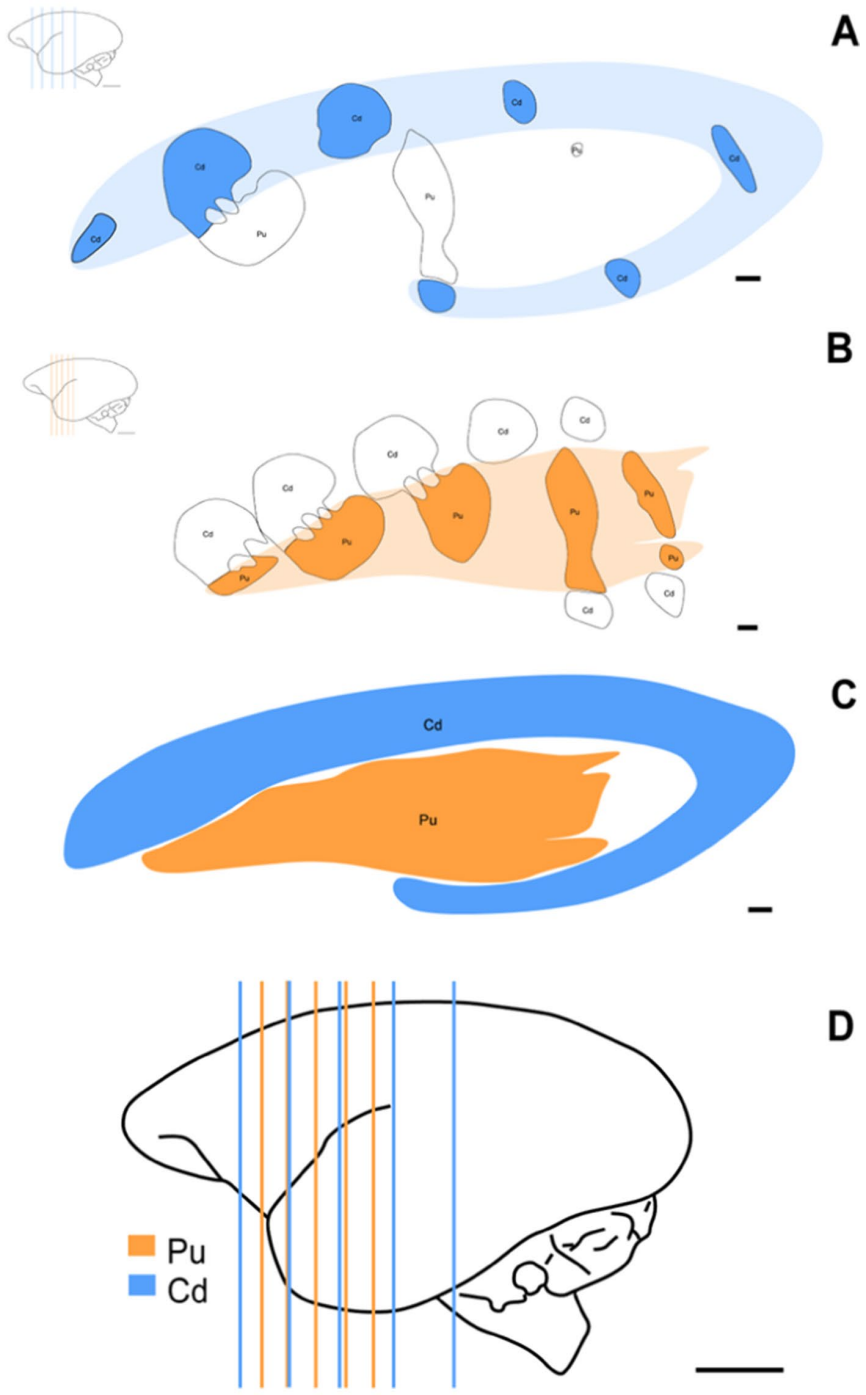
Schematic representation of the stereotaxic levels used and the three-dimensional morphology of St. Schematic of coronal sections showing the rostrocaudal arrangement of Cd **(A)** (AP: +1.30 mm to +13.30 mm) and Pu **(B)** (AP: +5.80 mm to +12.50 mm) at the approximate levels used for this study. **(C)** Schematic representation of the three-dimensional morphology of St, Cd, and Pu. **(D)** Brain distribution of simultaneously used stereotaxic levels of Cd and Pu. Scale bar in **(A–C)**: 1 mm. Scale bar in **(D)**: 5 mm.

Nissl staining was used to collect stereological data. A previous study of the material was conducted to establish optimal parameters for section fraction, square area count, grid spacing size, and dissector height. These stereological parameters were established based on Paxinos et al. (2012) and the evaluation of two experienced researchers. Considering the rostrocaudal arrangement of Cd and Pu, a random, uniform, and systematic sampling was conducted to obtain five sections for evaluating each nucleus separately, totaling the analysis of 10 sections per animal. Canvas 12 software was used to highlight the contours of the stereotaxic levels (Figure 2).

**Figure 3:**
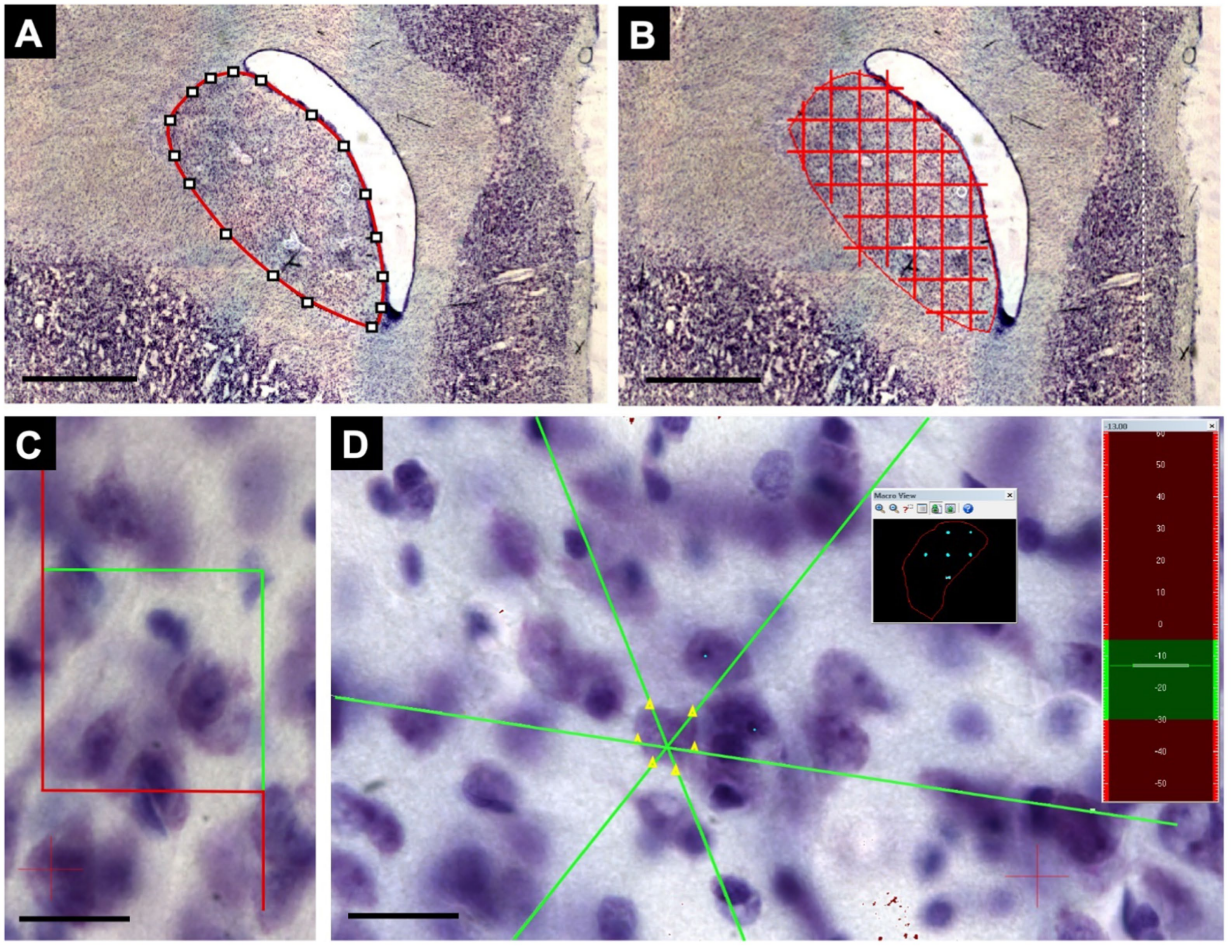
Steps for collecting stereological data. **(A)** Demarcation of the structure of interest using the lowest magnification objective, 2x, represented by a Cd level. **(B)** After applying the quadratic test system, the points within the nuclear area are counted, considering the same point inclusion criteria. Following Cavalieri’s principle, as adapted by Gundersen and Jensen, this is repeated for all sections to apply the sum of points obtained in the formulas. **(C)** Using the same demarcation of the nucleus, the optical dissector of 40x40μm was defined at the highest magnification (63x). **(D)** The software, through the optical fractionator, randomizes collection samples within the demarcated area (black square), where the count can be performed, and the neuronal volume can also be verified through the nucleolus and neuronal borders by the Nucleator tool using the Z axis observed in the sidebar (green and red). Scale bar in **(A,B)**: 1000 μm. Scale bar in **(C,D)**: 20 μm.

Analyses were performed using a Zeiss Axio Imager-Z2 microscope with a motorized stage integrated into the camera and a stereology apparatus with Stereo Investigator v11.0 software (MBF Bioscience) (Figure 3). The volume of each core region was estimated using Cavalieri’s principle, as adapted by Gundersen and Jensen (Gundersen and Jensen, 1987). Total striatal volume was measured by summing the estimated volumes for the Cd and Pu nuclei. The optical fractionator method was used to calculate the total number of neurons in the areas of interest (West et al., 1991) with 63× magnification (1.4 numerical aperture). Neuronal volume was estimated using the Nucleator tool based on the Rotator method (Jensen and Gundersen, 1993). Neuronal identification was performed using a detailed neuronal characteristics algorithm, and calibration was performed using the Neurocytology Test tool (García-Cabezas et al., 2016).

### 2.5 Data analysis

The data were tabulated and subjected to descriptive statistical analysis. Statistical analysis was performed using GraphPad Prism software (version 7.0). For neuronal volume analysis, each neuron was treated as a sampling unit. The Shapiro–Wilk test was used to verify the data distribution. Because the data did not meet the normality criteria, the Kruskal-Wallis test was applied. This robust, non-parametric statistical approach allows for comparing medians between groups, even when the data distribution is not normal. Both tests were conducted with a significance level set at *p* ≤ 0.05.

## 3 Results

### 3.1 Nissl-stained results

**Figure 4:**
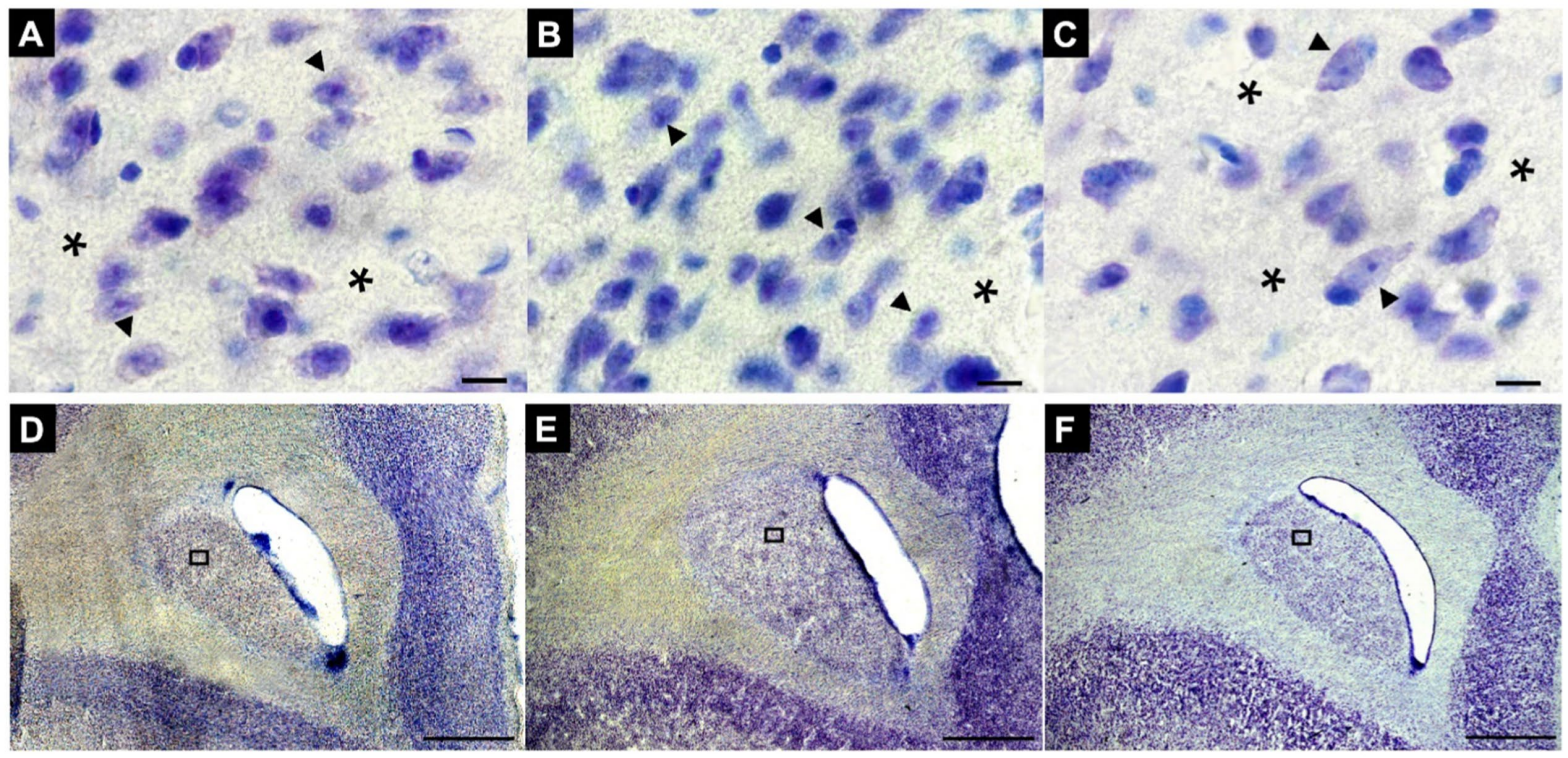
Representative photomicrographs of changes in neuronal volume, number and density in the St of animals FG **(A)**, IG **(B)**, and AG **(C)**, respectively. Note the conspicuous neuronal volume reduction and densities in B compared to A and C. Sections were obtained in the first rostral section of the Cd of animals in the FG **(D)**, IG **(E)** and AG **(F)**, respectively. ▶Arrowheads indicate neurons, and *asterisks indicate perineuronal regions. In **(A–C)**: Magnification 100x. Scale bar: 10 μm. In **(D–F)**: Magnification 2x. Scale bar: 1000 μm.

Sections of the striatal complex from juvenile marmosets subjected to social isolation protocols were stained using the Nissl method and subsequently analyzed through stereological techniques. Representative sections from the FG, IG, and AG groups were used (Figures 4D–F, respectively). Notably, a marked reduction in cell volume can be observed in the IG group (arrowheads) (Figure 4B) compared to the cells in the FG and AG groups (arrows) (Figures 4A,C, respectively).

### 3.2 Neuronal volume

An average of 175 neurons were sampled per animal. The mean neuronal volume of striatal neurons in the FG was 888.5 ± 349.1 μm^3^. The IG exhibited a significantly lower mean neuronal volume of 568.1 ± 279.5 μm^3^, while the AG showed a mean volume of 989.3 ± 478.3 μm^3^.

**Figure 5:**
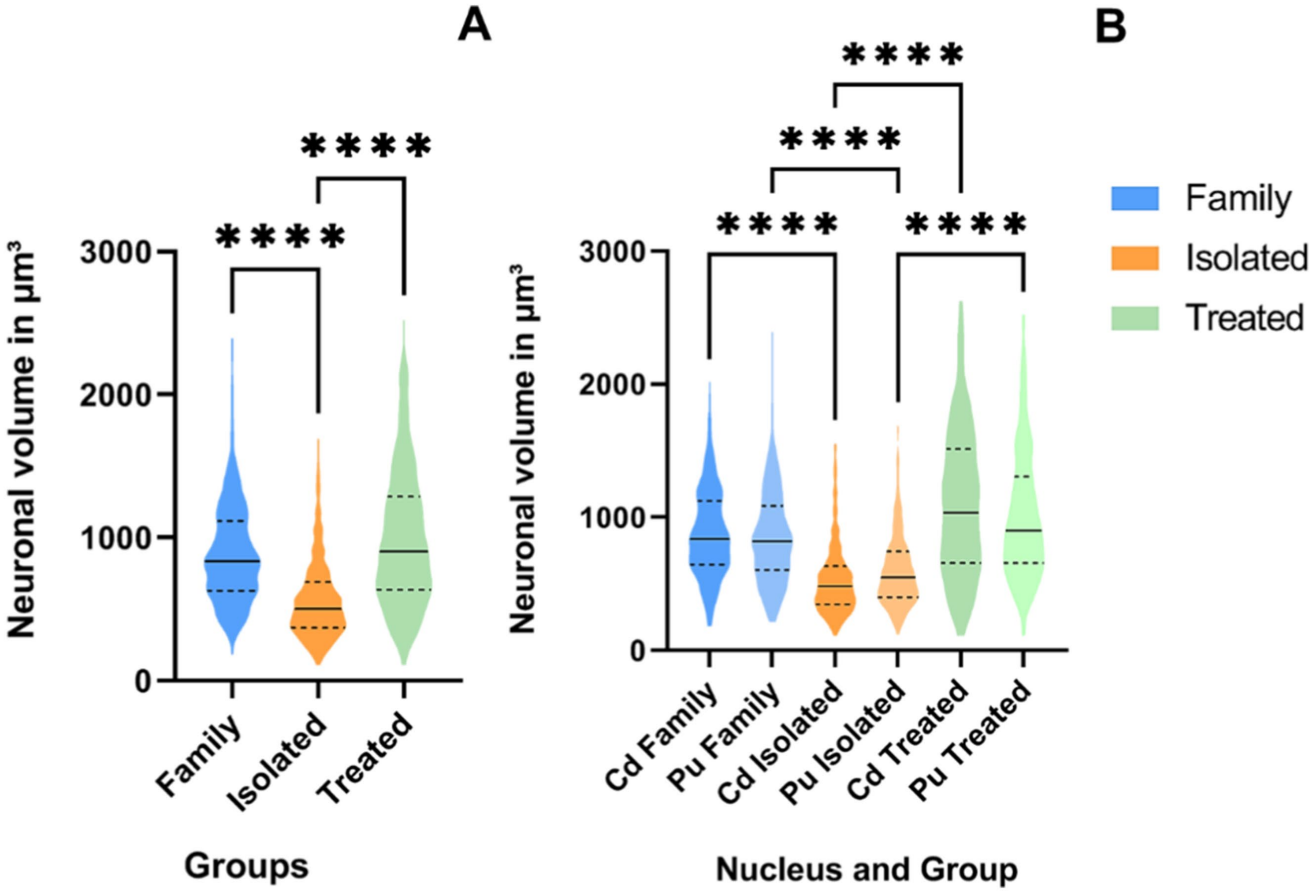
ViolinPlots of the comparison of neuronal volume in μm^3^ on the St **(A)** and per nucleus **(B)**. On the graph, a solid line represents the median, while dashed lines represent interquartile ranges. Comparisons using the Kruskal-Wallis test and Dunn’s *post-hoc* test. Significance levels are indicated by asterisks: ****p < 0.0001.

The Shapiro–Wilk test indicated a non-normal distribution of neuronal volume data (*p* < 0.001). Therefore, a Kruskal-Wallis test with Dunn’s *post-hoc* analysis was performed. The Kruskal-Wallis test revealed a significant difference in striatal neuron volume between the groups (*χ*^2^ = 256.2, *p* < 0.0001). Dunn’s *post-hoc* tests demonstrated that the FG and AG had significantly larger neuronal volume than the IG (*p* < 0.0001). No significant difference was observed between AG and FG (*p* = 0.6570) (Figure 5A). Notably, when comparing individually the nuclei, the volumes of the Cd and Pu in the FG and AG were significantly larger than their counterparts in the IG (*p* < 0.0001) (Figure 5B).

### 3.3 Neuronal density, estimated volume of nuclei, and estimated number of neurons

**Figure 6:**
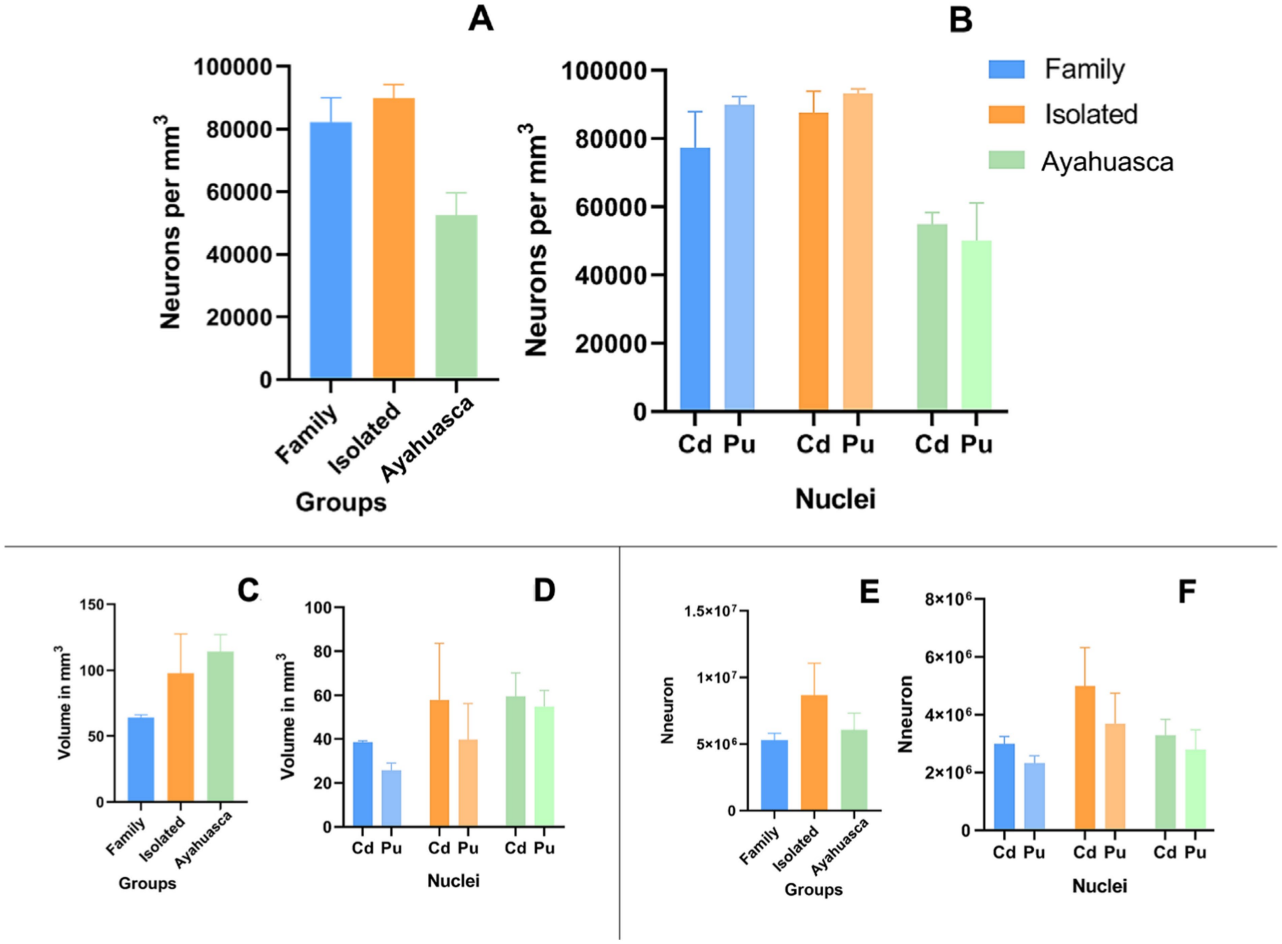
Column plots of neuronal distribution, estimated nuclear volume, and estimated neuronal number. Mean of neuronal distribution per mm^3^ on the St **(A)** and per nucleus **(B).** Mean of the estimated volume in mm^3^ on the St **(C)** and per nucleus **(D)**. Mean of the estimated number of neurons on the St **(E)** and on Cd and Pu **(F)**.

Neuronal density (neurons per mm^3^) was calculated by dividing the estimated number of neurons by the corresponding volume. Data from both animals in each group were included in the analysis. Figure 6 shows the mean values for the two animals in each group. The IG showed a higher neuronal density (89,838 ± 3,123 neurons/mm^3^) compared to the FG (82,301 ± 5,452 neurons/mm^3^) and the AG (52,539 ± 5,046 neurons/mm^3^). However, it is important to note that the small number of animals per group made it impossible to perform statistical inference tests, which merely represent trends.

Additionally, the estimated total striatal volume in the AG group (114.5 ± 12.76 mm^3^) showed a trend toward an increase compared to the IG group (97.68 ± 29.90 mm^3^) and the FG group (64.36 ± 1.90 mm^3^) (Figures 6C,D) with a calculated mean error coefficient of 3.2% (see Supplementary Table 1). Furthermore, ayahuasca treatment appears to have a prophylactic effect on estimated total striatal neuronal numbers, as the AG (6,078,000 ± 1,248,000) maintained approximately the same number of neurons as the FG (5,307,000 ± 507,000), in contrast to the IG (8,682,000 ± 2,382,000) (Figures 6E,F).

## 4 Discussion

Adolescents experience high rates of depression, which negatively impact cognitive development, leading to poor academic performance, substance abuse, behavioral problems, family conflicts, and suicides (Hauenstein, 2003; Frankish et al., 2018; Stringaris and Vidal-Ribas, 2019). Adolescence represents a critical period for brain development (Fuhrmann et al., 2015), and depression can exert a profoundly detrimental effect on neural maturation (Sacchet et al., 2016). Consequently, there is an urgent need to advance neurobiological research aimed at investigating the pathophysiology of depression and developing new approaches to alleviate the symptoms of this debilitating condition.

In a previous publication, we observed an apparent prophylactic effect of ayahuasca in preventing depressive-like behaviors and stimulating cortisol reactivity during social isolation (de Meiroz Grilo et al., 2022). In that study, male marmosets treated with ayahuasca exhibited a more adaptive stress response compared to isolated animals, along with fecal cortisol levels similar to those animals living in family groups.

The present study utilized the same brain samples from de Meiroz Grilo et al. (2022) to conduct a neuroanatomical investigation aimed at identifying potential structural changes in the marmoset striatum, as chronic stress and neuropsychiatric disorders are known to disrupt striatal circuits. For example, chronic stress can impair communication between somatostatin-positive interneurons and striatal medium-spiny neurons, leading to striatal overactivation, disinhibition, and increased motor output (Rodrigues et al., 2022). Furthermore, alterations in the glutamine-glutamate-GABA cycle between the striatum, hippocampus, and cerebellum may also contribute to the effects of chronic stress (Xu et al., 2020).

Neuroanatomical studies involving experimental models exposed to stress protocols are predominantly based on global MRI analyses conducted in humans. However, quantitative data at the cellular level related to depression in non-human primates remain unavailable, highlighting a critical gap in the literature.

Adolescents suffering from depression exhibit significant structural changes within their brains (Redlich et al., 2018; Pagliaccio et al., 2020; Wu et al., 2020). A recent MRI study found increased volume in the cortical areas and cerebellum of adolescents experiencing depression (Fu et al., 2023). Conversely, MRI studies in adults with major depressive disorder have reported increased volumes in subcortical regions, including the striatum, amygdala, and nucleus accumbens (Qi et al., 2018; Avnioglu et al., 2021). A study conducted with adolescents and young adults found greater gray matter volume in the dorsolateral prefrontal cortex of those with depression and lower gray matter volumes in the hippocampus compared to healthy controls (Straub et al., 2019).

Given the scarcity of morphoquantitative data on the effects of stress on neural structures in juvenile individuals, particularly at the cellular level, efforts to identify morphological changes associated with widely studied functional alterations provide a valuable opportunity to deepen our understanding of the underlying pathological mechanisms. At the same time, such investigations may assist in identifying prophylactic agents capable of mitigating the effects of the disease.

In our previous study de Meiroz Grilo et al. (2022), we observed reduced autogrooming behavior in AG animals, comparable to that of FG animals—a behavior associated with stress reduction. Similarly, FG and AG animals exhibited comparable scores for sucrose solution intake, which were significantly different from those of IG animals, indicating symptoms related to anxiety and depression in the IG group. Finally, animals in the AG group showed cortisol levels similar to those in the family control group. Taken together, the behavioral data from our previous work and the morphological data presented here appear to converge, supporting the prophylactic effect of ayahuasca in attenuating symptoms of chronic stress.

Overall, our findings indicate that neurons in the striatum of animals treated with ayahuasca maintained volumes (989.3 ± 478.3 μm^3^) comparable to those observed in animals kept in family groups (888.5 ± 349.1 μm^3^) and, interestingly, distinct from the neuronal volumes in isolated animals (568.1 ± 279.5 μm^3^). These results support our previous behavioral data, further reinforcing the potential prophylactic role of ayahuasca while highlighting its morphological effects on neural structures.

Conversely, neuronal number, density, and striatal volume did not show significant differences among the groups and cannot be compared statistically due to the limited number of animals. However, the AG group exhibited a trend toward reduced neuronal density compared to the other groups despite maintaining a neuronal number similar to that observed in the FG. Interestingly, animals treated with ayahuasca displayed larger striatal volumes, suggesting a potential compensatory process in the striatum.

A plausible explanation for these morphological changes could be related to glial responses to chronic stress induced by the social isolation protocol. Recent evidence from human postmortem brain tissue has shown that stress is associated with significant reductions in astrocytes across several brain regions (Lu et al., 2025). Further research is needed to deepen investigations and better understand the trend of increased striatal volume in animals treated with ayahuasca.

Although no statistical inference was observed in striatal volume, our data showed a relative increase in striatal volume, which aligns with recent findings in the literature. A recent MRI study conducted on depressed patients who attempted suicide revealed structural changes in the putamen (Pu) (Kang et al., 2020). Furthermore, depressed patients have been reported to exhibit striatal enlargement, particularly in the Pu region (Avnioglu et al., 2021).

Even though we have presented important findings, particularly regarding the prophylactic effect in preventing volumetric loss in striatal neurons, some limitations should be acknowledged. The first limitation concerns the number of animals available in each sample group. This factor should be carefully considered, as studies involving non-human primates typically face constraints related to sample size (Atapour et al., 2024; Teymornejad et al., 2024). This limitation affected the analysis of the collected data, particularly regarding nucleus volume, neuronal number, and neuronal densities. Although the histological changes observed *in situ* were striking—and additional animals would likely reveal significant statistical differences—further studies with larger sample sizes are necessary to strengthen these findings.

Secondly, the absence of females in the groups represents another limitation, especially considering the higher incidence of depression in women (Eid et al., 2019). Finally, the absence of sham gavages (e.g., with saline or water as a vehicle) in the stressed control group was primarily due to confinement restrictions imposed during the SARS-CoV-2 pandemic, which necessitated adjustments to the initial protocol.

To the best of our knowledge, this is the first study to examine the effects of social isolation on the striatum of non-human primates at the cellular level. Furthermore, it is the first to present consistent data on the prophylactic effects of classic psychedelic administration on the neuroanatomical cellular profiles of marmosets subjected to a social isolation protocol. Behavioral data indicate that ayahuasca, when administered prior to social isolation, attenuated symptoms of chronic stress (de Meiroz Grilo et al., 2022). Furthermore, an observational study comparing adolescent ayahuasca users and non-users found fewer psychiatric symptoms among users, suggesting a protective effect of ayahuasca (Da Silveira et al., 2005). The morphological evidence presented here supports ayahuasca’s prophylactic role, as striatal neurons maintained their volumes. These findings encourage future studies to further investigate the proposed prophylactic effect observed in striatal neurons.

## 5 Conclusion

Our findings suggest that ayahuasca may exert a prophylactic effect by preserving striatal neuron volumes and mitigating the effects of chronic stress induced by social isolation.

Behavioral data from our previous study demonstrated stress-reducing effects, including preserved cortisol levels and reduced depressive-like behaviors in treated animals. Although no inferential statistics were performed to compare neuronal density, trends suggest a potential reorganization of striatal nuclei in ayahuasca-treated animals, possibly linked to glial responses.

Morphological evidence highlights increased striatal volumes in ayahuasca-treated animals, aligning with recent literature on structural brain changes associated with depression. While the sample size and absence of female subjects may slightly limit the generalizability of these findings, the data presented here provide valuable insights that warrant further investigation to confirm ayahuasca’s prophylactic potential and to better understand its effects on neural structures.

## Data Availability

The raw data supporting the conclusions of this article will be made available by the authors, without undue reservation.

## References

[ref1] Alexander G. E. Crutcher M. D. (1990). Functional architecture of basal ganglia circuits: neural substrates of parallel processing. Trends Neurosci. 13, 266–271. doi: 10.1016/0166-2236(90)90107-L, PMID: 1695401

[ref2] Atapour N. Rosa M. G. P. Bai S. Bednarek S. Kulesza A. Saworska G.. (2024). Distribution of calbindin-positive neurons across areas and layers of the marmoset cerebral cortex. PLoS Comput. Biol. 20:e1012428. doi: 10.1371/journal.pcbi.1012428, PMID: 39312590 PMC11495585

[ref3] Avnioglu S. Velioglu H. A. Cankaya S. Yulug B. (2021). Quantitative evaluation of brain volumes in drug-free major depressive disorder using MRI-cloud method. Neuroreport 32, 1027–1034. doi: 10.1097/WNR.0000000000001682, PMID: 34075004

[ref4] Basile G. A. Bertino S. Bramanti A. Ciurleo R. Anastasi G. P. Milardi D.. (2021). Striatal topographical organization: bridging the gap between molecules, connectivity and behavior. Eur. J. Histochem. EJH 65:3284. doi: 10.4081/ejh.2021.3284, PMID: 34643358 PMC8524362

[ref5] Carbonaro T. M. Gatch M. B. (2016). Neuropharmacology of N,N-dimethyltryptamine. Brain Res. Bull. 126, 74–88. doi: 10.1016/j.brainresbull.2016.04.016, PMID: 27126737 PMC5048497

[ref6] Choi K. Holly E. N. Davatolhagh M. F. Beier K. T. Fuccillo M. V. (2019). Integrated anatomical and physiological mapping of striatal afferent projections. Eur. J. Neurosci. 49, 623–636. doi: 10.1111/ejn.13829, PMID: 29359830 PMC6056328

[ref7] Cipriani A. Furukawa T. A. Salanti G. Chaimani A. Atkinson L. Z. Ogawa Y.. (2018). Comparative efficacy and acceptability of 21 antidepressant drugs for the acute treatment of adults with major depressive disorder: a systematic review and network meta-analysis. Lancet 391, 1357–1366. doi: 10.1016/S0140-6736(17)32802-7, PMID: 29477251 PMC5889788

[ref8] da Silva F. S. Silva E. A. S. de Sousa G. M. Maia-de-Oliveira J. P. de Soares-Rachetti V. de Araujo D. B.. (2018). Acute effects of ayahuasca in a juvenile non-human primate model of depression. Braz. J. Psychiatry 41, 280–288. doi: 10.1590/1516-4446-2018-0140, PMID: 30427388 PMC6804303

[ref9] Da Silveira D. X. Grob C. S. de Rios M. D. Lopez E. Alonso L. K. Tacla C.. (2005). Ayahuasca in adolescence: a preliminary psychiatric assessment. J. Psychoactive Drugs 37, 129–133. doi: 10.1080/02791072.2005.10399792, PMID: 16149324

[ref10] de Castro Leão A. Neto D. D. Bernardete Cordeiro de Sousa M. (2009). New developmental stages for common marmosets (*Callithrix jacchus*) using mass and age variables obtained by K-means algorithm and self-organizing maps (SOM). Comput. Biol. Med. 39, 853–859. doi: 10.1016/j.compbiomed.2009.05.009, PMID: 19651403

[ref11] de Meiroz Grilo M. L. P. de Sousa G. M. de Mendonça L. A. C. Lobão-Soares B. de Sousa M. B. C. Palhano-Fontes F.. (2022). Prophylactic action of ayahuasca in a non-human primate model of depressive-like behavior. Front. Behav. Neurosci. 16:901425. doi: 10.3389/fnbeh.2022.901425, PMID: 36408451 PMC9672345

[ref12] de Sousa M. B. C. de Meiroz Grilo M. L. P. Galvão-Coelho N. L. (2021). Natural and experimental evidence drives marmosets for research on psychiatric disorders related to stress. Front. Behav. Neurosci. 15:674256. doi: 10.3389/fnbeh.2021.674256, PMID: 34177478 PMC8227430

[ref13] Eid R. S. Gobinath A. R. Galea L. A. M. (2019). Sex differences in depression: insights from clinical and preclinical studies. Prog. Neurobiol. 176, 86–102. doi: 10.1016/j.pneurobio.2019.01.006, PMID: 30721749

[ref14] Frankish H. Boyce N. Horton R. (2018). Mental health for all: a global goal. Lancet 392, 1493–1494. doi: 10.1016/S0140-6736(18)32271-2 30314858

[ref15] Fu Y.-J. Liu X. Wang X.-Y. Li X. Dai L.-Q. Ren W.-Y.. (2023). Abnormal volumetric brain morphometry and cerebral blood flow in adolescents with depression. World J. Psychiatry 13, 386–396. doi: 10.5498/wjp.v13.i6.386, PMID: 37383288 PMC10294138

[ref16] Fuhrmann D. Knoll L. J. Blakemore S.-J. (2015). Adolescence as a sensitive period of brain development. Trends Cogn. Sci. 19, 558–566. doi: 10.1016/j.tics.2015.07.008 26419496

[ref17] Galvão A. C. M. de Almeida R. N. EADS S. FAM F. Palhano-Fontes F. Onias H.. (2018). Cortisol modulation by Ayahuasca in patients with treatment resistant depression and healthy controls. Front. Psych. 9:185. doi: 10.3389/fpsyt.2018.00185, PMID: 29867608 PMC5952178

[ref18] Galvão-Coelho N. L. Galvão A. C. D. M. Silva F. S. D. Sousa M. B. C. D. (2017). Common marmosets: a potential translational animal model of juvenile depression. Front. Psych. 8:175. doi: 10.3389/fpsyt.2017.00175, PMID: 28983260 PMC5613153

[ref19] Galvão-Coelho N. L. Silva H. P. A. de Leão A. de Sousa M. B. C. (2008). Common marmosets (*Callithrix jacchus*) as a potential animal model for studying psychological disorders associated with high and low responsiveness of the hypothalamic-pituitary-adrenal axis. Rev. Neurosci. 19, 187–201. doi: 10.1515/REVNEURO.2008.19.2-3.187, PMID: 18751524

[ref20] Ganzel B. L. Morris P. A. (2011). Allostasis and the developing human brain: explicit consideration of implicit models. Dev. Psychopathol. 23, 955–974. doi: 10.1017/S0954579411000447, PMID: 22018076

[ref21] García-Cabezas M. Á. John Y. J. Barbas H. Zikopoulos B. (2016). Distinction of neurons, glia and endothelial cells in the cerebral cortex: an algorithm based on cytological features. Front. Neuroanat. 10:107. doi: 10.3389/fnana.2016.00107, PMID: 27847469 PMC5088408

[ref22] Graybiel A. M. (2008). Habits, rituals, and the evaluative brain. Annu. Rev. Neurosci. 31, 359–387. doi: 10.1146/annurev.neuro.29.051605.112851, PMID: 18558860

[ref23] Gundersen H. J. Jensen E. B. (1987). The efficiency of systematic sampling in stereology and its prediction. J. Microsc. 147, 229–263. doi: 10.1111/j.1365-2818.1987.tb02837.x, PMID: 3430576

[ref24] Han S. Zheng R. Li S. Liu L. Wang C. Jiang Y.. (2023). Progressive brain structural abnormality in depression assessed with MR imaging by using causal network analysis. Psychol. Med. 53, 2146–2155. doi: 10.1017/S0033291721003986, PMID: 34583785

[ref25] Hankin B. L. (2006). Adolescent depression: Description, causes, and interventions. Epilepsy Behav. 8, 102–114. doi: 10.1016/j.yebeh.2005.10.012 16356779

[ref26] Harmer C. J. Duman R. S. Cowen P. J. (2017). How do antidepressants work? New perspectives for refining future treatment approaches. Lancet Psychiatry 4, 409–418. doi: 10.1016/S2215-0366(17)30015-9, PMID: 28153641 PMC5410405

[ref27] Hauenstein E. J. (2003). Depression in adolescence. JOGNN 32, 239–248. doi: 10.1177/0884217503252133, PMID: 12685676

[ref28] Ho R. C. M. Zhang M. W. (2016). Ketamine as a rapid antidepressant: the debate and implications. BJPsych Adv. 22, 222–233. doi: 10.1192/apt.bp.114.014274

[ref29] Hussain H. Dubicka B. Wilkinson P. (2018). Recent developments in the treatment of major depressive disorder in children and adolescents. BMJ Ment Health 21, 101–106. doi: 10.1136/eb-2018-102937, PMID: 30045844 PMC10270457

[ref30] Jensen E. B. V. Gundersen H. J. G. (1993). The rotator. J. Microsc. 170, 35–44. doi: 10.1111/j.1365-2818.1993.tb03321.x

[ref31] Kang W. Shin J.-H. Han K.-M. Kim A. Kang Y. Kang J.. (2020). Local shape volume alterations in subcortical structures of suicide attempters with major depressive disorder. Hum. Brain Mapp. 41, 4925–4934. doi: 10.1002/hbm.25168, PMID: 32804434 PMC7643352

[ref32] Kovich H. Kim W. Quaste A. M. (2023). Pharmacologic treatment of depression. Am. Fam. Physician 107, 173–181, PMID: 36791444

[ref33] Lee M. T. Peng W.-H. Kan H.-W. Wu C.-C. Wang D.-W. Ho Y.-C. (2022). Neurobiology of depression: chronic stress alters the glutamatergic system in the brain—focusing on AMPA receptor. Biomedicines 10:1005. doi: 10.3390/biomedicines10051005, PMID: 35625742 PMC9138646

[ref34] LeMoult J. Battaglini A. M. Grocott B. Jopling E. Rnic K. Yang L. (2023). Advances in stress and depression research. Curr. Opin. Psychiatry 36, 8–13. doi: 10.1097/YCO.0000000000000831 36194148

[ref35] Lu C.-L. Ren J. Cao X. (2025). An Astroglial basis of major depressive disorder: molecular, cellular, and circuit features. Biol. Psychiatry 97, 217–226. doi: 10.1016/j.biopsych.2024.07.017, PMID: 39084500

[ref36] Luan L. X. Eckernäs E. Ashton M. Rosas F. E. Uthaug M. V. Bartha A.. (2024). Psychological and physiological effects of extended DMT. J. Psychopharmacol. Oxf. Engl. 38, 56–67. doi: 10.1177/02698811231196877, PMID: 37897244 PMC10851633

[ref37] Marx W. Penninx B. W. J. H. Solmi M. Furukawa T. A. Firth J. Carvalho A. F.. (2023). Major depressive disorder. Nat. Rev. Dis. Primer 9:44. doi: 10.1038/s41572-023-00454-1, PMID: 37620370

[ref38] Miranda J. M. D. Valle R. Cruz M. A. O. M. Bezerra B. M. Valença-Montenegro M. M. Oliveira L., et al. (2015). *Callithrix jacchus* (amended version of 2018 assessment). *IUCN Red List Threat. Species*. Available at: https://www.iucnredlist.org/en (accessed June 10, 2024).

[ref39] Miura Y. Li M.-Y. Birey F. Ikeda K. Revah O. Thete M. V.. (2020). Generation of human striatal organoids and cortico-striatal assembloids from human pluripotent stem cells. Nat. Biotechnol. 38, 1421–1430. doi: 10.1038/s41587-020-00763-w, PMID: 33273741 PMC9042317

[ref40] Osório Fde L. Sanches R. F. Macedo L. R. Santos R. G. Maia-de-Oliveira J. P. Wichert-Ana L.. (2015). Antidepressant effects of a single dose of ayahuasca in patients with recurrent depression: a preliminary report. Rev. Bras. Psiquiatr. 37, 13–20. doi: 10.1590/1516-4446-2014-1496, PMID: 25806551

[ref41] Pachaly J. R. (2024). “Terapêutica por Extrapolação Alométrica” in Tratado de Animais Selvagens-Medicina Veterinária. eds. Cubas Z. S. Silva J. C. R. Catão-Dias J. L., vol. 2006 (São Paulo: Roca), 1215–1223.

[ref42] Pagliaccio D. Alqueza K. L. Marsh R. Auerbach R. P. (2020). Brain volume abnormalities in youth at high risk for depression: adolescent brain and cognitive development study. J. Am. Acad. Child Adolesc. Psychiatry 59, 1178–1188. doi: 10.1016/j.jaac.2019.09.032, PMID: 31634568 PMC7165045

[ref43] Palhano-Fontes F. Barreto D. Onias H. Andrade K. C. Novaes M. M. Pessoa J. A.. (2019). Rapid antidepressant effects of the psychedelic ayahuasca in treatment-resistant depression: a randomized placebo-controlled trial. Psychol. Med. 49, 655–663. doi: 10.1017/S0033291718001356, PMID: 29903051 PMC6378413

[ref44] Paxinos G. Watson C. Rosa M. Petrides M. Tokuno H. (2012). The marmoset brain in stereotaxic coordinates. New York, NY: Springer.

[ref45] Perkins D. Ruffell S. G. D. Day K. Pinzon Rubiano D. Sarris J. (2022). Psychotherapeutic and neurobiological processes associated with ayahuasca: A proposed model and implications for therapeutic use. Front. Neurosci. 16:879221. doi: 10.3389/fnins.2022.879221, PMID: 36798604 PMC9928213

[ref46] Provost J.-S. Hanganu A. Monchi O. (2015). Neuroimaging studies of the striatum in cognition part I: healthy individuals. Front. Syst. Neurosci. 9:140. doi: 10.3389/fnsys.2015.00140, PMID: 26500513 PMC4596942

[ref47] Pryce C. R. Rüedi-Bettschen D. Dettling A. C. Feldon J. (2002). Early life stress: long-term physiological impact in rodents and primates. News Physiol. Sci. Int. J. Physiol. Prod. Jointly Int. Union Physiol. Sci. Am. Physiol. Soc. 17, 150–155. doi: 10.1152/nips.01367.2001, PMID: 12136043

[ref48] Qi Q. Wang W. Deng Z. Weng W. Feng S. Li D.. (2018). Gray Matter Volume Abnormalities in the Reward System in First-Episode Patients with Major Depressive Disorder. Cham: Springer International Publishing, 704–714.

[ref49] Qin D. Chu X. Feng X. Li Z. Yang S. Lü L.. (2015). The first observation of seasonal affective disorder symptoms in Rhesus macaque. Behav. Brain Res. 292, 463–469. doi: 10.1016/j.bbr.2015.07.005, PMID: 26164484

[ref50] Redlich R. Opel N. Bürger C. Dohm K. Grotegerd D. Förster K.. (2018). The limbic system in youth depression: brain structural and functional alterations in adolescent in-patients with severe depression. Neuropsychopharmacology 43, 546–554. doi: 10.1038/npp.2017.246, PMID: 29039414 PMC5770774

[ref51] Rodrigues D. Jacinto L. Falcão M. Castro A. C. Cruz A. Santa C.. (2022). Chronic stress causes striatal disinhibition mediated by SOM-interneurons in male mice. Nat. Commun. 13:7355. doi: 10.1038/s41467-022-35028-4, PMID: 36446783 PMC9709160

[ref52] Rossi G. N. Guerra L. T. L. Baker G. B. Dursun S. M. Saiz J. C. B. Hallak J. E. C.. (2022). Molecular pathways of the therapeutic effects of Ayahuasca, a botanical psychedelic and potential rapid-acting antidepressant. Biomol. Ther. 12:1618. doi: 10.3390/biom12111618, PMID: 36358968 PMC9687782

[ref53] Sacchet M. D. Ho T. C. Connolly C. G. Tymofiyeva O. Lewinn K. Z. Han L. K.. (2016). Large-scale Hypoconnectivity between resting-state functional networks in Unmedicated adolescent major depressive disorder. Neuropsychopharmacology 41, 2951–2960. doi: 10.1038/npp.2016.76, PMID: 27238621 PMC5061890

[ref54] Savoldi R. Polari D. Pinheiro-da-Silva J. Silva P. F. Lobao-Soares B. Yonamine M.. (2017). Behavioral changes over time following Ayahuasca exposure in zebrafish. Front. Behav. Neurosci. 11:139. doi: 10.3389/fnbeh.2017.00139, PMID: 28804451 PMC5532431

[ref55] Shepherd G. M. G. (2013). Corticostriatal connectivity and its role in disease. Nat. Rev. Neurosci. 14, 278–291. doi: 10.1038/nrn3469, PMID: 23511908 PMC4096337

[ref56] Siemsen B. M. Franco D. Lobo M. K. (2022). Corticostriatal contributions to dysregulated motivated behaviors in stress, depression, and substance use disorders. Neurosci. Res. S0168-0102:304. doi: 10.1016/j.neures.2022.12.014, PMID: 36565858

[ref57] Straub J. Brown R. Malejko K. Bonenberger M. Grön G. Plener P. L.. (2019). Adolescent depression and brain development: evidence from voxel-based morphometry. J. Psychiatry Neurosci. JPN 44, 237–245. doi: 10.1503/jpn.170233, PMID: 30720261 PMC6606428

[ref58] Stringaris A. Vidal-Ribas P. (2019). Probing the irritability-suicidality Nexus. J. Am. Acad. Child Adolesc. Psychiatry 58, 18–19. doi: 10.1016/j.jaac.2018.08.014, PMID: 30577933

[ref59] Teymornejad S. Majka P. Worthy K. H. Atapour N. Rosa M. G. P. (2024). Bilateral connections from the amygdala to extrastriate visual cortex in the marmoset monkey. Cereb. Cortex 34:bhae348. doi: 10.1093/cercor/bhae348, PMID: 39227312

[ref60] Thapar A. Collishaw S. Pine D. S. Thapar A. K. (2012). Depression in adolescence. Lancet 379, 1056–1067. doi: 10.1016/S0140-6736(11)60871-4, PMID: 22305766 PMC3488279

[ref61] West M. J. Slomianka L. Gundersen H. J. (1991). Unbiased stereological estimation of the total number of neurons in the subdivisions of the rat hippocampus using the optical fractionator. Anat. Rec. 231, 482–497. doi: 10.1002/ar.1092310411, PMID: 1793176

[ref62] WHO (2023). Depressive disorder (depression). Available at: https://www.who.int/news-room/fact-sheets/detail/depression (Accessed June 10, 2024).

[ref63] Wu B. Li X. Zhou J. Zhang M. Long Q. (2020). Altered whole-brain functional networks in drug-Naïve, first-episode adolescents with major depression disorder. JMRI 52, 1790–1798. doi: 10.1002/jmri.27270, PMID: 32618061

[ref64] Xu S. Liu Y. Pu J. Gui S. Zhong X. Tian L.. (2020). Chronic stress in a rat model of depression disturbs the glutamine-glutamate-GABA cycle in the striatum, Hippocampus, and cerebellum. Neuropsychiatr. Dis. Treat. 16, 557–570. doi: 10.2147/NDT.S245282, PMID: 32158215 PMC7047974

