# Supplementary material for: Ayahuasca partially preserves striatal integrity in juvenile non-human primates exposed to chronic stress: evidence from stereological evaluation
Source: Front Neuroanat. 2025 Feb 19;19:1457557. doi: 10.3389/fnana.2025.1457557 (PMC11886963; doi:10.3389/fnana.2025.1457557)
Supplement: Supplementary file 1 [file Table_1.docx]

**Supplementary Material**

Table of the Error Coefficient calculated per animal and per nucleus

| Animal | Cd | Pu |
| --- | --- | --- |
| FG1 | 3,29% | 2,86% |
| FG2 | 3,48% | 3,78% |
| IG1 | 3,01% | 3,28% |
| IG2 | 3,03% | 3,08% |
| TG1 | 3,36% | 2,71% |
| TG2 | 3,07% | 2,97% |
